# Targeting Metalloenzymes: The “Achilles’ Heel” of Viruses and Parasites

**DOI:** 10.3390/ph16060901

**Published:** 2023-06-19

**Authors:** Dimitrios Moianos, Georgia-Myrto Prifti, Maria Makri, Grigoris Zoidis

**Affiliations:** Department of Pharmacy, Division of Pharmaceutical Chemistry, School of Health Sciences, National and Kapodistrian University of Athens, Panepistimiopolis Zografou, 15771 Athens, Greece; moianosjim@gmail.com (D.M.); myrtoprifti@gmail.com (G.-M.P.); mariamakri1@hotmail.com (M.M.)

**Keywords:** metalloenzymes, metal chelators, antiviral agents, antiparasitic agents, influenza A, HBV, HCV, HIV, *Trypanosoma brucei*, *Trypanosoma cruzi*

## Abstract

Metalloenzymes are central to the regulation of a wide range of essential viral and parasitic functions, including protein degradation, nucleic acid modification, and many others. Given the impact of infectious diseases on human health, inhibiting metalloenzymes offers an attractive approach to disease therapy. Metal-chelating agents have been expansively studied as antivirals and antiparasitics, resulting in important classes of metal-dependent enzyme inhibitors. This review provides the recent advances in targeting the metalloenzymes of viruses and parasites that impose a significant burden on global public health, including influenza A and B, hepatitis B and C, and human immunodeficiency viruses as well as *Trypanosoma brucei* and *Trypanosoma cruzi*.

## 1. Introduction

Metal ions are essential for the proper functioning of numerous proteins, and thus these proteins are commonly known as metalloproteins. Approximately one-third of all proteins are believed to be metalloproteins [1,2,3], and some of them are responsible for crucial viral and parasitic functions [4,5]. In metalloproteins, the role of the metal ion can be either structural (e.g., zinc-finger proteins) or functional. A metalloprotein can be classified as a metalloenzyme when the metal ion it contains plays a role in promoting catalysis [6]. Even though the vast majority of metalloenzyme inhibitors rely on coordinate bonds between the inhibitor and the active site metal center as a critical interaction, the range of functional groups utilized in inhibitors to create these important interactions is surprisingly limited. Typically, metalloenzyme inhibitors are small drug-like molecules that bear various metal-binding groups (MBGs). These MBGs contain appropriate atoms (typically O or N) that can coordinate with the metal ions present in the active site of the enzyme. The MBG is usually linked to the “backbone” of the inhibitor, which is the drug-like part, through an appropriate linker [7]. The most frequently used MBG is hydroxamic acid followed by reverse hydroxamates, carboxylates, thiols, and phosphinates.

The backbone portion, which is the domain that recognizes the target, is intricate and varies depending on the active site of the enzyme. Adjusting the backbone of the inhibitor to match the metalloenzyme’s active site can enhance its effectiveness and specificity against the target. Additionally, although metalloenzymes are ubiquitous, the number of FDA-approved therapeutics that target them as validated targets is relatively small. This represents an extensive and unexplored target space.

The aim of this review is to present the latest advancements in targeting metalloenzymes that play a crucial role in the pathogenesis of specific diseases. Our particular emphasis is on metal-chelating agents with antiviral and antiparasitic properties. We have chosen to focus on viruses and parasites that we believe impose a significant burden on global public health, including influenza A and B, hepatitis B and C, and human immunodeficiency viruses as well as *Trypanosoma brucei* and *Trypanosoma cruzi*. We begin by briefly outlining the role of each metalloenzyme in the diseases’ pathogeneses, highlighting the most notable chemical agents that target metalloenzymes. Finally, we present the latest compounds that modulate the activity of the selected metalloenzymes.

## 2. Influenza Virus RNA-Dependent RNA Polymerase PA N-Terminal Endonuclease Domain

Influenza viruses affect the respiratory system and cause annual epidemics that result in substantial morbidity and mortality, especially among adults aged 65 years and older and people with underlying health conditions [8,9]. Considerably, recent estimates show that influenza infections are the cause of 300,000 to 500,000 deaths [10,11,12] and over 5 million hospitalizations annually [13], making it a severe burden for global health worldwide. Vaccination remains the best way to prevent influenza outbreaks, although vaccines should be annually updated and have reduced effectiveness due to the constant antigenic drifts and occasional antigenic shifts that make the virus able to evade the host immune response [14,15,16]. The currently available anti-influenza drugs include neuraminidase inhibitors oseltamivir, zanamivir, and peramivir and polymerase inhibitors baloxavir and favipiravir (which is only approved for use in humans in Japan) [17,18,19]. Adamantane derivatives amantadine and rimantadine, which block M2 ion channel activity, are no longer used due to the emergence of drug-resistant virus strains [20].

Influenza viruses contain a single-stranded minus-polarity RNA genome in complex with RNA-dependent RNA polymerase (RdRP) [21]. Each RNA segment is attached to a single RdRP molecule. RdRP is a heterotrimeric protein comprised of three subunits, the PA (polymerase acidic protein) and PB1 and PB2 (polymerase basic proteins 1 and 2, respectively), and is responsible for the viral RNA replication [22]. However, the virus cannot generate the necessary 5′-mRNA cap, and it therefore uses the host’s 5′-capped mRNA sequences and cleaves the generated RNA 8–14 nucleotides downstream. This process is known as “cap-snatching” and is catalyzed by the PA subunit. The resulting mRNA is elongated by the PB2 subunit and is recognizable by the host cell factors [23,24,25,26]. The PA contains two subunits: the *N*-terminal domain (PA_N_) contains the endonuclease active site, and the C-terminal domain plays a structural role [27]. The influenza RdRP, particularly the metal-dependent PA_N_ endonuclease domain of the enzyme, is an attractive target for new influenza antiviral agents, as it is essential for the viral lifecycle and is highly conserved among influenza strains [28]. Baloxavir is the only FDA-approved PA_N_ endonuclease inhibitor and was introduced to the market in 2018. However, a mutation in the viral genome that replaces Ile 38 with Thr in the PA_N_ (I38T) grants the virus resistance to baloxavir and reduces its anti-influenza effectiveness [29].

Previous efforts have identified potent PA_N_ inhibitors that bear chemically diverse MBGs and act by chelating the metal cations in the enzyme catalytic site. Those include polyphenol natural products, such as epigallocatechin gallate, the phenylethylcatechol derivatives of thalidomide, diketo acids (DKAs), flutamide, 2,3-hydroxyquinolones, 5,6-hydroxypyrimidinones, 5-bromo-2,3-hydroxypyridinones, and 3,4-hydroxypyridinones, which were reviewed by Chen et al. [1].

**Figure 1:**
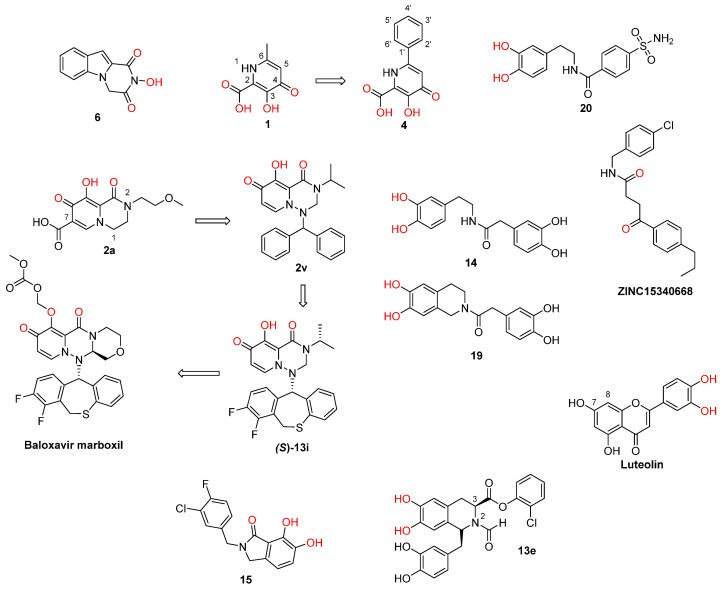
Structures of reported influenza PA_N_ inhibitors. Arrows indicate the optimization process of lead compounds **1** and **2a** to yield compound **4** (and its analogs) and Baloxavir marboxil, respectively. Metal-chelating atoms are highlighted in red.

**Figure 2:**
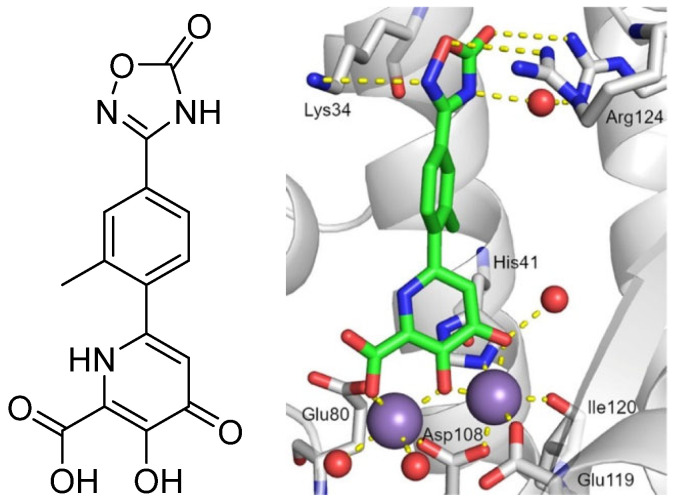
Interactions of compound **24** with key amino acids of the PA_N_ active site (PDB: 6E4C). Compound **24** chelates the divalent cations of the active site and forms solvent-mediated or direct hydrogen bonding interactions with Lys34, Arg124, and/or Arg196. Mn^2+^ ions and water molecules are shown with purple and red spheres, respectively. Metal coordination and hydrogen bonds are represented by dashed yellow lines. Reprinted/adapted with permission from Ref. [32]. Copyright © 2023, American Chemical Society.

Recent research findings have elucidated additional knowledge towards the objective of attaining more potent inhibition of the enzyme. In 2016, our research group undertook the design and synthesis of a range of flutimide analogues that incorporated the indole 2,6-diketopiperazine moiety into the established pharmacophore of the indole. The resulting molecules displayed remarkable activity in PA_N_ enzymatic assays (IC_50_ values as low as 12.7 μM for compound **6**, Figure 1). Nevertheless, their efficacy was comparatively lower in cellular experiments, probably due to poor cell permeability [30]. In 2018, Credille et al. employed in vitro endonuclease and cell assays as well as X-ray crystallography to explore the SAR of several well-known MBGs including hydroxypyrone, hydroxypyridinone, hydroxypyrimidine, and hydroxytropolone. Their results highlight the importance of the electronic and steric characteristics of the inhibitors with respect to both the activity and selectivity of the related metalloenzymes. They also displayed that different metalloenzymes exhibit very distinct preferences regarding these ligand characteristics at their binding sites [31]. One of the most potent inhibitors identified in this campaign, pyridinone **1** (Figure 1, IC_50_ = 17 nM), was further explored by the same group to establish even more precise SARs. More specifically, they incorporated a phenyl ring in the 6-position of the pyridine ring of **1** to produce lead compound **4** (Figure 1), and various substitutions on the phenyl ring were introduced, to generate derivatives of **4**. Through this process, it was found that small alkyl substituents in the 2’ and acidic substituents in the 4’ phenyl ring position as well as a combination of these were the most favorable for achieving a potent action, resulting in compounds with IC_50_ values below 2.5 nM and with no significant cytotoxicity. The generated analogs were also selective for PA_N_ inhibition over similar metalloenzymes. They also utilized X-ray crystallography to identify the key interactions between the most potent compounds and the key amino acids in the enzyme binding site. Importantly, as illustrated by the crystal structure of compound **24** in the PA_N_ active site, acidic substituents are favorable due to their antiviral activity, as they form hydrogen bonding interactions with Lys34, Arg124, and/or Arg196 (Figure 2). Nevertheless, these compounds gave poor results in cell assays, likely due to poor cell permeability [32].

**Figure 3:**
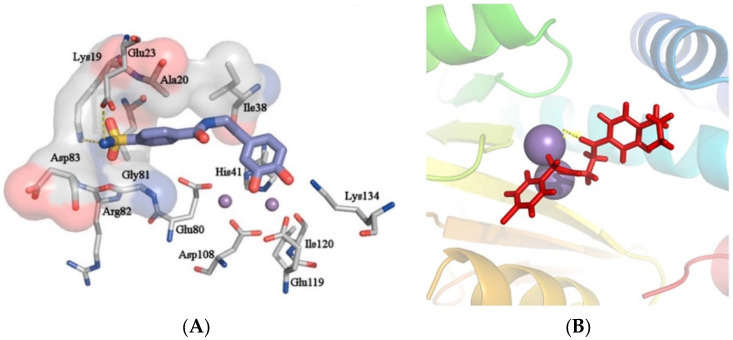
Predicted poses of Compound **20** (**A**) and ZINC15340668 (**B**) in the PA_N_ binding site. In both images, divalent cations are presented as purple spheres. Reprinted/adapted with permission from Ref. [33]. Copyright © 2023, Elsevier, [34]. Copyright © 2023, Elsevier.

Another group employed computational methods to establish a 3D pharmacophore model for the PA_N_ endonuclease. Through virtual screening and docking studies, they identified compound **20** (Figure 1), a sulfonamide analogue that bears a catechol moiety. The docking studies utilized the previously described crystal structure of the enzyme cocrystallized with epigallocatechin-3-gallate and illustrated that the two catechol OH groups of **20** are able to chelate the two Mg^2+^ ions of the binding site, while the sulfonamide group interacts through hydrogen bonding with the key amino acids Glu23 and Lys19 (Figure 3A). Compound **20** proved potent in enzymatic PA_N_ assays, showcasing the model’s effectiveness. This particular analog can serve as a lead for chemical optimization to further improve its interactions with the key residues in the enzyme’s binding site while retaining metal chelation [33]. Nevertheless, it remains to be determined whether compound **20** retains its activity in cellular experiments, as the sulfonamide group may limit its membrane permeability. Molecular docking and molecular dynamics were also used by Mohseni et al. to screen a large number of compounds from the ZINC library that exhibited similarity with a known inhibitor. The highest-scoring compound, **ZINC15340668** (Figure 1), was shown to interact with the key binding site amino acids through hydrophobic interactions while chelating one of the two cations present in the enzyme’s binding site (Figure 3B). It was also tested against the I38T-mutant enzyme and proved able to inhibit the mutant enzyme with high efficiency. The same compound illustrated exceptional theoretical ADME properties and is therefore suitable to serve as a primer for further in vivo and in vitro experiments with the ultimate aim of validating these promising but theoretical characteristics [34].

**Figure 4:**
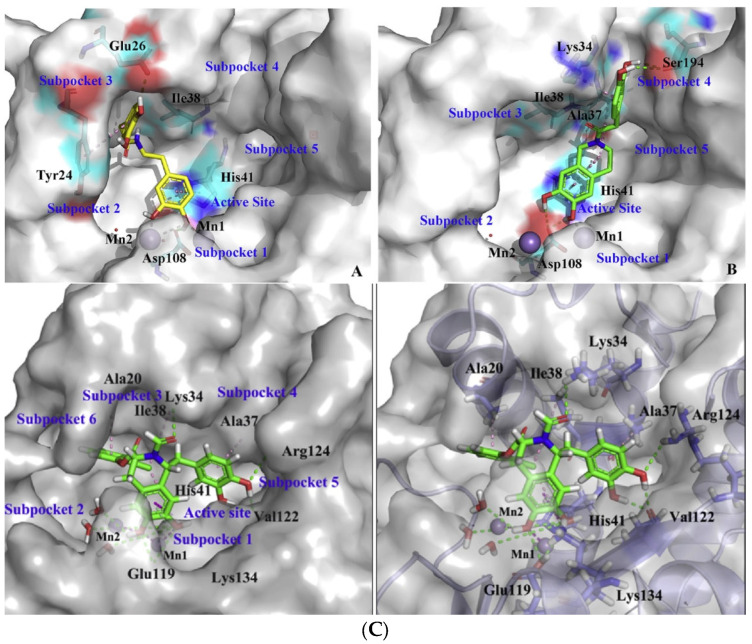
Binding pose of compounds **14** (**A**), **19** (**B**), and **13e** (**C**) in PA_N_ binding site according to docking calculations. Divalent cations are presented as purple spheres. Reprinted/adapted with permission from Ref. [38] Copyright © 2023, Elsevier and [42] Copyright © 2023, Elsevier.

Miyagawa et al. set to explore the SARs on the known hit compound **2a** (Figure 1), which bears a carbamoyl pyridine bicycle (CAB). They identified the more potent CAB analogue **2v** (Figure 1), which had improved characteristics, including reasonable pharmacokinetic properties, and was also effective in mouse models [35]. The N-1 side chain and N-3 substitution on the CAB ring were further optimized, resulting in compound **(*S*)-13i** (Figure 1), which contained a N1-dihydrobenzothiepine ring and exhibited exceptional results in mouse models. The optimization of this compound led to the approved PA_N_ inhibitor baloxavir marboxil [36]. Ivashchenko et al. designed and synthesized analogues of baloxavir bearing more flexible groups, resulting in the novel inhibitor **AV5124**, which showed less potency against baloxavir-resistant strains (I38T) than the wild-type viral strains [37]. Liao et al. designed and synthesized potent anti-influenza A dopamine conformationally constrained analogs, namely, 1,2,3,4-tetrahydroisoquinoline-6,7-diols, and explored their SARs. The most potent compounds **14** and **19** (Figure 1) were proven to bind with the influenza PA_N_, and they both displayed properties comparable to those of the known inhibitor peramivir. Their research also included a molecular docking analysis, which concluded that the two OH groups of the dopamine moiety of **14** or the 1,2,3,4-tetrahydroisoquinoline-6,7-diol moiety of **19** are essential for the activity, as they chelate the two Mg^2+^ ions in the binding site (Figure 4A,B). Furthermore, the nature of the amide linker and the substituents in the phenyl ring played a crucial role in the antiviral activity [38]. Reiberger et al. explored the C-7 and C-8 derivatives of the previously identified PA_N_ flavonoid inhibitor luteolin (Figure 1) [39]. They introduced multiple substitutions in both the C-7 and the C-8 positions of the luteolin ring and tested the antiviral activity of the newly synthesized analogs in vitro. The conversion of the C-7 OH of luteolin to other substituents failed to increase the potency, while the C-8 position could tolerate different substituents while retaining the antiviral activity [40]. Further experiments are, however, required, in order to assess the possible therapeutic potential of this class of PA_N_ inhibitors. Rogolino et al. explored the 2,3-dihydroisoindole scaffold. More specifically, they kept the 2,3,-dihydroisoindole ring containing the three oxygen atoms that chelate the two Mg^2+^ ions in the binding site intact and altered the substitution in the lipophilic moiety linked to the MBG. They obtained analogs that inhibited the PA_N_ as well as the I38T-mutant PA_N_ with IC_50_ values in the nM range in enzymatic assays, with the most potent being 3-chloro-4-fluorobenzyl *N*-substituted analogue **15** (Figure 1, IC_50_ = 24.6 nM). Some of the compounds were also potent in cell-based influenza assays, while none of them were cytotoxic. However, the activity was diminished in cell- compared to enzyme-based experiments, probably due to low cell permeability. Molecular docking was also used to identify the binding mode of this class of compounds and their key interactions with the binding site. Importantly, the two OH groups and the carbonyl group chelate the Mg^2+^ cations in a way that is similar to DKA metal chelators. [41]. Liu et al. optimized the known 6,7-dihydroxy-1,2,3,4-tetrahydroisoquinoline PA_N_ inhibitor scaffold and managed to obtain the new inhibitor **13e** (Figure 1), which contains an amide substitution in the C-2 position and a halogenated phenyl ester group in the C-3 position of the core scaffold and is potent in both enzymatic (EC_50_ = 0.28 μM) and cell assays (IC_50_ = 4.50 μM) while not showing cytotoxicity (CC_50_ > 100 μM). The binding mode of **13e** in the PA_N_ enzyme was also elucidated through docking studies (Figure 4, C) [42]. These favorable characteristics highlight that this class of analogs can be explored further through structural modifications, with the ultimate aim of developing potent PA_N_-inhibiting agents with therapeutic applications.

Overall, several chemically distinct PA_N_ inhibitors, able to chelate the divalent cations present in its binding site, were recently identified. However, with the exception of the approved baloxavir marboxil, no other compound has managed to enter clinical testing. The biggest limitation of the above-mentioned inhibitor classes is their poor ability to penetrate cell membranes, which results in the loss of their activity in cell assays. On the other hand, most of them show very promising PA_N_ inhibition at the enzymatic level. Therefore, research must focus on addressing the cell permeability issue, probably through prodrug formulations—following the example of baloxavir marboxil—or isostere substitutions. Furthermore, as with all antiviral agents, the emerging viral resistance to baloxavir highlights the need to develop novel inhibitors that combat baloxavir-resistant I38T viral strains effectively.

## 3. Hepatitis B Virus—HBV Polymerase (Ribonuclease H)

Hepatitis B virus is a member of the *Hepadnaviridae* family, and it affects the liver and causes hepatitis B disease. It has been categorized into ten distinct genotypes (A to J) [43,44]. The mode of transmission includes both exposure to infectious blood or other body fluids (e.g., semen and vaginal secretions—sexual intercourse) and perinatal exposure from infected mothers to infants [16]. Remarkably, as per the World Health Organization’s (WHO) records, chronic HBV infections affect over 250 million individuals and result in over 800,000 deaths annually, with SE Asian, African, and Western Pacific countries showing the highest epidemic prevalence [20,22,23]. Hepatitis B is divided into acute and chronic infection, with the chronic one considered to be one of the foremost causes of hepatic cirrhosis as well as the primary etiological agent responsible for hepatocellular carcinoma. The availability of a safe and effective vaccine remains the most suitable way to prevent hepatitis B outbursts, although it appears that this cannot fully avert the disease. The antiviral agents that are presently employed, including pegylated interferon and nucleos(t)ide analogues, such as adefovir, lamivudine, tenofovir, and entecavir, which block HBV reverse transcriptase activity and therefore HBV DNA replication, displayed significant limitations and are unable to achieve complete eradication of the virus from infected cells [44,45,46,47].

HBV polymerase is the enzyme responsible for HBV replication, and has two enzymatic properties. As a reverse transcriptase, the enzyme synthesizes the DNA from an RNA template and simultaneously, as a RNaseH, degrades this RNA template, the viral pgRNA. Its RNaseH active site features four conserved carboxylates that bind two essential Mg^+2^ ions [48,49]. The aforementioned carboxylates constitute a “DEDD” (aspartic acid–glutamic acid–aspartic acid–aspartic acid) motif, which coordinates the two Mg^2+^ ions. Neither of the two ions could be omitted due to their importance in the RNA hydrolysis process [50,51]. More precisely, ribonuclease H enzymes catalyze the RNA sequences’ cleavage in DNA:RNA hybrids and are included in the general category of endonuclease enzymes [52,53]. Consequently, inhibition of the HBV RNaseH enzymatic activity results in the synthesis of noninfectious HBV virions, that contain defective genome [54].

The basic structural characteristic of all known HBV RNaseH inhibitors is that they all bear three electron donors (O or N), the so-called triad, that chelate the two Mg^2+^ ions in the enzymes’ active sites [55]. The first identified chemical categories of RNaseH inhibitors were the α-hydroxytropolones (α-HTs) and *N*-hydroxyimides followed by the *N*-hydroxyisoquinolinediones (HIDs), *N*-hydroxynapthyridines (HNOs), *N*-hydroxypyridinediones (HPDs), and N-hydroxypyrimidinediones [48,55,56,57]. **β-thujaplicinol** is one of the initially discovered HBV RNaseH inhibitors, as it blocks the RNaseH of viral genotypes D and H, with EC_50_ values of 5.9 and 2.3 μΜ, respectively. It is classified as α-HT, and its origin lies in the heartwood of western red cedar [58]. This compound became the lead for the design and synthesis of several new hydroxylated tropolones that combat HBV replication, with very low EC_50_ values (as low as 0.34 μM), CC_50_ values up to 100 μΜ and therapeutic indexes up to 200 (Table 1) [55,59,60]. Further structure–activity relationship studies in the α-HT ring revealed that the α-OH substitution is necessary for the inhibition of the HBV RNaseH. Sulfonyl or lactone substituents on the tropolone ring enhance the inhibitory activity, while bulky substitutions in positions R^1^, R^2^, and R^3^ decrease it. This has also been validated in several recent studies [59,61,62,63,64].

Three additional chemotypes, specifically the HIDs, HPyDs, and HNOs, consisting of a hydroxyimide or *N*-hydroxyimide-like moiety are able to inhibit the HBV RNaseH [48,55,65]. All these categories contain, as mentioned, O or N atoms in the appropriate positions to chelate the two Mg^2+^ ions, similar to the α-HTs [55]. The distinction between the HPyD and HID scaffolds and the HNO scaffold is that the first ones possess an oxygen trident, which is required for antiviral activity, while the trident is absent from the HNOs, where one oxygen is replaced by an aromatic nitrogen. This nitrogen has a lone pair of electrons which is accessible for magnesium cation coordination [55,66]. Following the screening of many analogues against the HBV RNaseH, low EC_50_ values, high therapeutic indexes, and limited cytotoxicity were confirmed. In fact, one of the *N*-hydroxyimide analogues, compound **208**, was studied in in vivo experiments, and the great activity of these analogues against the virus was verified [48,56,65,66,67]. The chemical structures of several analogues recognized as HBV RNaseH inhibitors are presented in Table 1.

## 4. Hepatitis C Virus Nonstructural Protein 5B (NS5B)

Hepatitis C is a communicable illness that gradually harms the liver, ultimately leading to cirrhosis and death. It is a severe public health issue globally, affecting up to 3% of the population and resulting in over 300,000 deaths annually [68,69,70]. Its slow progression and challenging detection make it a concealed pandemic, and the majority of infections result in long-lasting conditions that can persist for years. Roughly 60–80% of individuals infected with HCV have persistent hepatitis, with about 20% developing cirrhosis and 2–5% succumbing to liver cirrhosis and malignancy [71,72]. In 2019, there were about 58 million severe documented cases worldwide, with 75% of them occurring in low- and middle-income countries [73]. New HCV infections are emerging in high-risk populations globally, such as drug users [74]. Sub-Saharan Africa is primarily affected by HCV transmission through unsafe medical procedures and infected blood transfusions. Additionally, needlestick injuries in healthcare professionals, mother-to-child transmission, and societal behaviors such as piercing and tattooing may serve as potential transmission routes for the virus [74,75].

Currently, in the treatment of HCV infections, direct-acting antivirals primarily focus on nonstructural proteins NS3/4A, NS5A, and NS5B [76]. Of these, NS5B is a vital enzyme responsible for the synthesis of HCV RNA strands. As an RdRP, NS5B utilizes the original RNA chain as a template and facilitates the polymerization of ribonucleoside triphosphates (rNTPs) to generate new RNA chains [77,78,79]. The NS5B protein comprises three domains: the fingers, thumb, and palm regions [78,80]. The active center of NS5B, situated in the palm region, is accountable for the catalysis of the nucleophilic reaction between the 3′-terminal hydroxyl group of the RNA extension chain and the rNTP substrates [81]. The central structure of the active site is stabilized by a chelating complex composed of two Mg^2+^ ions and conserved amino acid residues D220, D318, and D319 [78].

Several metal ion chelators with varying structural compositions have demonstrated significant inhibitory effects against NS5B, including α,γ-DKAs [82], meconic acids [83], 5,6-dihydroxypyrimidine-4-carboxylic acids [84,85], and 2-hydroxyisoquinoline-1,3-diones [86]. According to molecular simulations, these compounds may interact with the two Mg^2+^ ions located in the active site of NS5B via a “tridentate” chelation mechanism [85,86]. However, most metal ion chelators reported to exhibit anti-HCV activity have not achieved the anticipated results at the cellular level. This may be due to the presence of carboxyl-containing metal-chelating functional groups, which result in the low membrane permeability of the compounds. It is therefore evident that the characteristics of the metal-chelating inhibitors described before necessitate further optimization [86].

**Figure 5:**
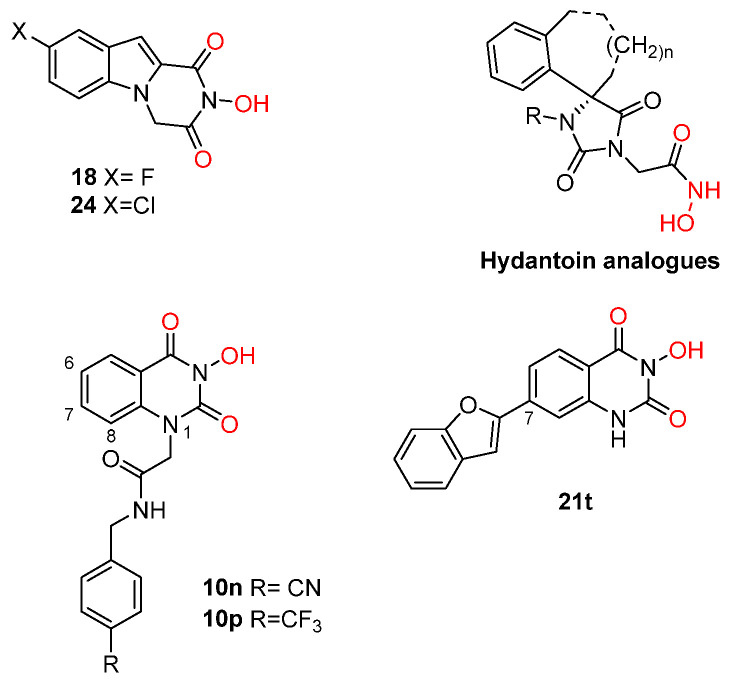
Structures of reported HCV NS5B inhibitors. Metal-chelating atoms/groups are highlighted in red.

**Figure 6:**
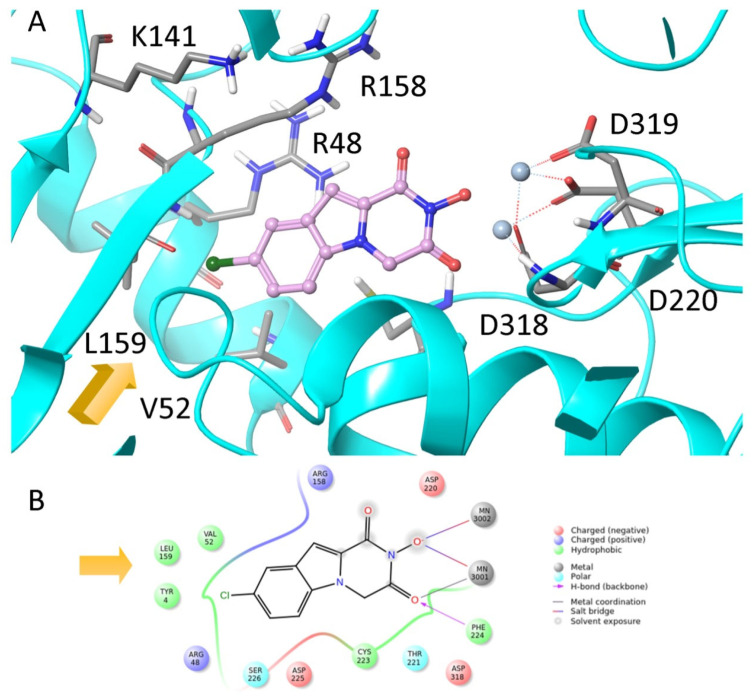
(**A**) Predicted binding orientation of compound **24** in the catalytic site of HCV polymerase. (**B**) Two-dimensional diagram of the interactions between the inhibitor and the polymerase’s active site. Docking illustrated the existence of a lipophilic cavity (indicated with yellow arrows) adjacent to the enzyme catalytic pocket. Reprinted/adapted with permission from Ref. [30]. Copyright © 2023, Royal Society of Chemistry.

The above-mentioned PA_N_-inhibiting flutimide analogues (Section 2), designed and synthesized by our group, act as dual inhibitors, as they not only block the influenza PA_N_ endonuclease but also the HCV NS5B enzyme. Our investigations identified two specific compounds, namely, **18** and **24** (Figure 5), both featuring halogens on the indole ring. These compounds demonstrated fair potency against the HCV strains (EC_50_ values of 83.8 μM and 10.5 μM, respectively) while simultaneously exhibiting low levels of toxicity (LC_50_ values > 200 μM), resulting in good selectivity index values. Further analysis through docking studies revealed that the indole group of these compounds was directed towards a lipophilic region within the active site, while two of three oxygen atoms effectively chelated with the two Mg^2+^ ions (Figure 6) [30]. These findings suggest that the core scaffold could be subjected to further optimization in order to increase potency and selectivity through more direct interactions with the important amino acids present in the NS5B binding site. The indole-flutamide group was subjected to further investigation by our research team. Through its combination with established antitrypanocidal 2,6-diketopiperazine acetohydroxamic acids, we were able to synthesize compounds (hydantoin analogs) with heightened potency, exhibiting dual efficacy not only against HCV but also against two *Trypanosoma* species (Section 6) [87]. A more recent study by Cao et al. explored the 3-hydroxyquinazoline-2,4(1H,3H)-dione scaffold. The group introduced different substituents to the N-1 position of the core scaffold and the C-6, C-7, and C-8 positions as well as combinations of these. Their findings suggest that amides bearing phenyl substituents in the N-1 position are favorable for their activity, which are represented by compounds **10n** (Figure 5, EC_50_ = 6.4 μΜ) and **10p** (Figure 5, EC_50_ = 8.1 μΜ). Furthermore, a C-7 aryl substitution on the parent compound significantly improved the antiviral activity. More specifically, when the phenyl ring at C-7 contains an electron-withdrawing group, the toxicity is decreased. Moreover, the phenyl furan substitution at C-7 yields compound **21t** (Figure 5), which displays the lowest EC_50_ among the currently known anti-HCV metal chelators (2.0 μΜ). Finally, the metal-chelating mechanism of action of the synthesized 3-hydroxyquinazoline-2,4(1H,3H)diones was predicted through molecular docking experiments [88].

## 5. *Trypanosoma brucei* 6-Oxopurine Phosphoribosyltransferase (PRT)

Human African trypanosomiasis (HAT), also known as African sleeping sickness, is a severe and often lethal disease caused by the protozoan parasite *Trypanosoma brucei*. The disease is endemic to 36 countries in Sub-Saharan Africa, with an estimated 20,000 new cases and 65 million people at risk of infection according to the World Health Organization [89,90]. The disease is spread to humans through the bite of an infected tsetse fly. Infected patients gradually progress to a coma and severe organ failure, which is usually fatal [91]. There are currently five therapeutic agents available for the treatment of HAT, namely, pentamidine, eflornithine, nifurtimox, melarsoprol, and suramin. However, these treatments have several severe side effects, including hypoglycemia, hypotension, encephalopathic syndrome, peripheral neuropathy, and hepatic toxicity, and they are becoming less effective due to emerging drug resistance [91,92,93]. In particular, the rate of treatment failure with melarsoprol has been reported to be as high as 39% in the last decade [92,94,95]. Eflornithine is also not an ideal therapy due to its requirement for intravenous administration [91,92,93,94]. Therefore, new targets within the *Trypanosoma brucei* parasite must be identified to develop new and more effective treatments for this neglected disease [96].

*T. brucei* is a unicellular eukaryotic parasite that relies heavily on purine salvage for its survival. The 6-oxopurine phosphoribosyltransferase (PRT) enzyme is an essential enzyme in the purine salvage pathway of *T. brucei* that is responsible for the conversion of 6-oxopurines into their corresponding nucleotides. The gene encoding the 6-oxopurine PRT enzyme has been identified and characterized, and biochemical studies have revealed important insights into its enzymatic mechanism and kinetics. The mechanism of action of the 6-oxopurine PRT enzyme involves metal chelation [97]. Upon binding to the active site, the substrate undergoes a nucleophilic attack on the alpha-phosphorus of 5-phosphoribosyl-1-pyrophosphate (PRPP), leading to the formation of the nucleotide monophosphate product and the release of pyrophosphate. The metal ion is thought to play a crucial role in the transfer of the pyrophosphate from the substrate to the PRPP, thereby stabilizing the transition state and promoting the reaction. Metal chelation is a crucial aspect of this mechanism, as it enables the enzyme to maintain its structure and catalytic activity [98,99]. Therefore, the 6-oxopurine PRT enzyme is a potential target for drug development against trypanosomiasis, and inhibitors that disrupt the enzyme’s metal chelation could be promising therapeutic agents.

**Figure 7:**
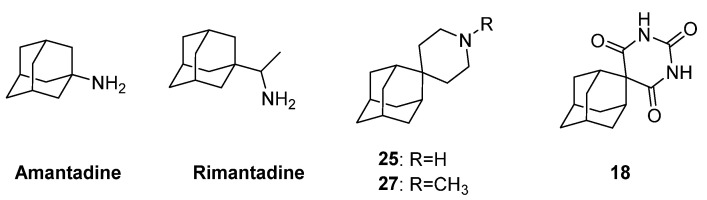
The active adamantane spiro derivatives and amantadine and rimantadine, two known antiviral agents against influenza A.

In recent years, allegations have been made that *T. brucei* is susceptible to anti-influenza A drugs rimantadine and amantadine. This led to extended research on aminoadamantane derivatives and the optimization of their metal-chelating pharmacophore. Hydrophobicity plays a crucial role in potency against both *Trypanosoma* species, *T. brucei* and *T. cruzi*. Consequently, the early reports include spiro-piperidine-4,2′-adamantane derivatives that resemble rimantadine with the addition of a lipophilic domain. Numerous bioactivity assays were conducted using bloodstream-form *T. brucei*, resulting in one active compound. Piperidine **25** (Figure 7) was 100% potent against parasites. In fact, it was proven to be 1.5-fold more active than rimantadine and at least 25-fold more potent than amantadine. Additionally, compound **18**, a barbituric analogue (Figure 7), killed 61% of the parasites demonstrating a moderate potency compared to rimantadine; however, it was seven times more potent than amantadine. Those findings did not suggest a metal chelation mechanism of action, but they played a crucial role in the following investigations, as compound **18** was the basis for the design of the next generation of molecules [100].

**Figure 8:**
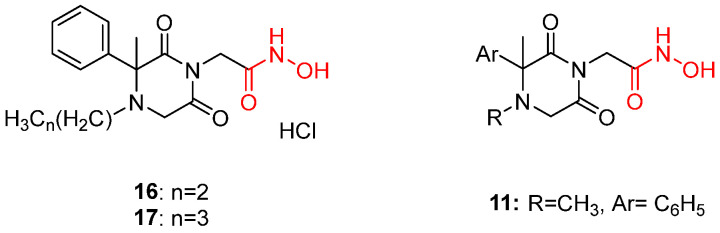
Compounds **16**, **17,** and **11**. Some of the most promising agents against *T. brucei* parasites. Metal-chelating atoms/groups are highlighted in red.

**Figure 9:**
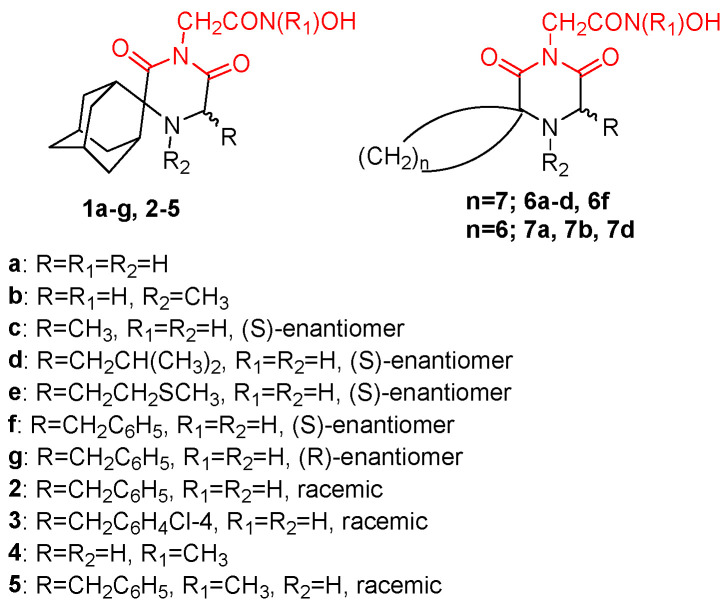
Spiro carbocyclic 2,6-DKP-1-acetohydroxamic acid analogues. Metal-chelating atoms/groups are highlighted in red.

More recent studies are focusing on the original hypothesis based on the barbituric analogue **18**. Modifying the basic structure of these molecules resulted in spiro 2,6-diketopiperazine (2,6-DKP) derivatives combined with acetohydroxamic moieties in the imidic nitrogen [101]. This class of compounds proved to be a valuable scaffold as a lead, and the hydroxamic unit seems indispensable due to its potency. Consequently, these findings led to the assumption that hydroxamic acid derivatives act through the chelation of the metal ion in the catalytic site of a metalloenzyme vital for the *T. brucei* parasites. Extended SARs on this scaffold revealed the importance of a benzyl group next to the amine nitrogen for both *T. brucei* and *T. cruzi* antiparasitic potency [102]. A variety of compounds was evaluated. To identify the exact parameters necessary for inhibiting the growth of *T. brucei* parasites, nonconstrained 3-alkyl-3-aryl-2,6-DKP molecules were designed and synthesized. The most active compound of this class was *N*-4-methyl derivative **11** (Figure 8) with a submicromolar IC_50_ (0.55 μΜ) followed by compounds **16** and **17** (Figure 8), which were remarkably selective against *T. brucei* parasites [101]. Following these findings, another active analogue, namely, (S)-chiral **1f** (Figure 9), bearing a benzyl substitution exhibited an IC_50_ value of 6.8 nM against *T. brucei*. This molecule was reported to be a potent inhibitor of not only *T. brucei* but also *T. cruzi* parasites. The ablation of the hydroxamic acid unit demonstrated the loss of trypanocidal activity, proving that this moiety is responsible for the chelation with metal ions in the catalytic center of the enzyme [102].

All in all, the future direction of the efforts aimed at discovering new antiviral agents that specifically target *T. brucei* parasites and address the challenge of treating sleeping sickness involves the design and evaluation of new molecules that combine the acetohydroxamic acid unit, which has already been demonstrated to have great antitrypanocidal activity, with a lipophilic tail resembling the antiviral flutimide pharmacophore [87]. Although the number of findings in the literature concerning the metal chelator inhibitors of the 6-oxopurine PRT enzyme is limited, the molecules that have been reported thus far bearing metal-chelating groups (such as acetohydroxamic moieties) show promising results and significant activity against *T. brucei* parasites, whereas, in the absence of these substitutions, the antitrypanocidal potency is lost. [49].

**Figure 10:**
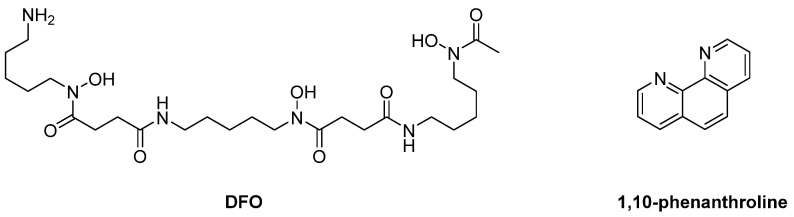
DFO and 1,10-phenanthroline were the first reported iron-chelating inhibitors of iron-dependent enzymes in *T. brucei* species.

*T. brucei* bloodstream forms have only four iron-dependent enzymes: alternative oxidase, aconitase, superoxide dismutase, and ribonucleotide reductase. This means that the ablation of iron through chelation could inhibit their normal activity and lead to parasite death. Moreover, *T. brucei* species do not have an iron storage protein like mammalian cells, and, as a consequence, there would be no other way to replace the iron needed for those four enzymes [103]. Another strategy to design potent metal-chelating inhibitors against *T. brucei* is the synthesis of iron-binding agents. Iron plays a crucial role in the immune system and in the pathogenesis of all parasites, such as *Trypanosoma*. Thus, compounds capable of forming complexes with iron could lead to its depletion and could therefore lead to a decrease in trypanocidal DNA synthesis and an increase in oxidative stress levels. Multiple studies have reported molecules of this type with promising results, although the exact mechanism of action is not fully understood yet. There have been reports on 13 molecules tested for their metal-chelating properties and antitrypanocidal activity against *T. brucei* species. Among them, five demonstrated significant IC_50_ values in the micromolar range: desferrioxamine B (DFO); the 2,9-dimethy-4,7-diphenyl analogue of bathocuproine, which was previously reported to be a copper chelator; 1,10-phenanthroline and its 4,7-diphenyl analogue; and 8-hydroxyquinoline, which is also supposed to be an iron chelator. All five compounds demonstrated potential antitrypanocidal activity. However, they inhibited human HL-60 cells, leading to a moderate therapeutic index. Future research efforts should prioritize evaluating the selectivity of these molecules as well as obtaining a more comprehensive understanding of their chelating mechanism. Notably, **DFO** and **1,10-phenanthroline** (Figure 10) were the most selective and the least cytotoxic analogs. This study highlights that future antitrypanocidal agents must exhibit a substantial degree of lipophilicity [104,105].

**Figure 11:**
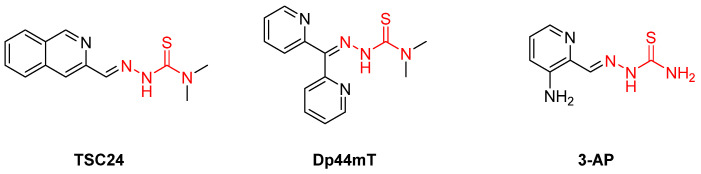
Three new generation iron chelators of four iron-dependent T. brucei enzymes. Metal-chelating atoms/groups are highlighted in red.

A more recent study reported three iron chelators from the thiosemicarbazone family. **TSC24**, **Dp44mT**, and **3-AP** (Figure 11) were found to inhibit *T. brucei* parasites at a minimum inhibition concentration of 1–100 μΜ and at acceptable 50% growth inhibition (GI_50_ = 250 nM) values. All three exhibited similar trypanotoxic activity, but 3-AP seems to act differently from the others. Adding iron to the examined cells showed a decrease in the trypanocidal potency of TSC24 and Dp44mT, but this was not the case for 3-AP, suggesting that the latter has a dual mechanism of action [106]. Although TSC24 and Dp44mT inhibit the iron-dependent enzymes through iron chelation, 3-AP seems to destruct a tyrosyl radical of ribonucleotide reductase [107]. However, all three demonstrated a possible dual mechanism, as they inhibited topoisomerase IIα too. Whether this feature acts synergistically with iron chelation for antitrypanocidal activity remains elusive. The most important finding of this study is the essential thiosemicarbazone scaffold, which is responsible for the iron chelation mechanism of action. Despite their promising results, their selectivity was poor and remains an issue to be addressed in future research [106].

## 6. *Trypanosoma cruzi* Carbonic Anhydrase (CA)

Chagas disease, also known as American trypanosomiasis, is a significant neglected illness that predominantly affects Latin America and is caused by a protozoan parasite called *Trypanosoma cruzi* (*T. cruzi*) which is transmitted to humans via the bite of a triatomine bug [108,109]. Due to migration, the disease can be found in nonendemic areas, such as Europe and the United States, affecting an estimated 11 million people worldwide [110]. However, only two drugs, benznidazole and nifurtimox, have been approved for treating the infection, both of which are frequently associated with unpleasant side effects and low effectiveness [111,112]. Therefore, there is a requirement to discover new compounds with trypanocidal activity that are safer and more effective than the current drugs [113,114].

*T. cruzi* employs a complex replication cycle involving multiple stages and intricate molecular mechanisms. During the intracellular amastigote stage, *T. cruzi* depends on carbonic anhydrase (CA) to maintain pH homeostasis, which is essential for survival and proliferation within the host cell. CA catalyzes the reversible conversion of carbon dioxide and water into bicarbonate and protons, contributing to the regulation of the intracellular pH, which is critical for many cellular processes. The mechanism of action of *T. cruzi* carbonic anhydrase (CA) involves the coordination of a zinc ion with three histidine residues, which form the active site of the enzyme. The zinc ion assists in the binding and activation of the substrate, CO_2_, by facilitating the transfer of a proton from the zinc-bound water molecule to the substrate. The coordinated zinc ion is held in place by the three histidine residues and a water molecule, forming a tetrahedral complex. The coordination of the zinc ion with the histidine residues is crucial for the enzymatic activity of CA, as the mutation of any of these residues results in a loss of catalytic activity. Additionally, the chelation of the zinc ion by CA has been found to be crucial for its stability and proper folding as well as its resistance to denaturation by chaotropic agents. The metal chelation property of CA has been exploited for the design of potent inhibitors, which bind to the zinc ion and prevent its coordination with the active site residues. These inhibitors exhibit antitrypanosomal activity and may provide a potential avenue for the development of new drugs for the treatment of Chagas disease [115,116,117,118,119].

As soon as CA was fully characterized, several studies explored already known and novel molecules that could inhibit the α-CA found in *T. cruzi*. This isoform, named TcCA, has a Zn^+2^ in its catalytic site which forms complexes with three histidine amino acids and a water molecule [117]. The inhibition of this enzyme by sulfonamides, thiols, anions, hydroxamates, and sulfamates with significant trypanocide potency in a nanomolar range in vitro has been reported [115].

**Figure 12:**
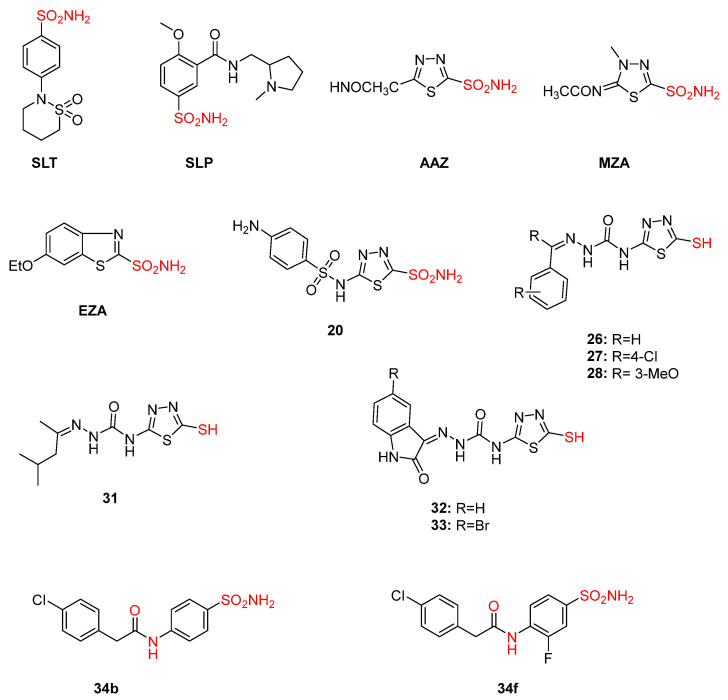
Chemical structures of AAZ, MZA, SLT, SLP, and EZA, which are known from literature, and novel sulfonamide inhibitors examined for their trypanocidal activity with promising results. Metal-chelating atoms/groups are highlighted in red.

Sulfonamides, first discovered in 1935 by Domagk, were used as antimicrobial, antiobesity, and diuretic agents. Their ability to bind with metal ions gave the green light for further investigation against Trypanosome species [120]. Indeed, several molecules from those studies resulted in clinical drugs, such as acetazolamide (AAZ), dichlorphenamide (DCP), ethoxzolamide (EZA), and methazolamide (MZA), that have been used until recently as classic TcCA inhibitors (Figure 12). Compound **20**, derived from this first study, along with sulpiride–sulthiame (**SLP-SLT**) proved to be the best candidates for maximum potency, with an IC_50_ of 61.6–93.6 nM against *T. cruzi* (Figure 12). The disadvantage of this class of compounds is that sulfonamides cannot easily penetrate cell membranes, resulting in poor in vivo potency [119]. Thus, following studies reported the introduction of a mercapto moiety that is usually ionized and is capable of chelating metals such as zinc cations. Similar to the SO_2_NH^−^ group that binds Zn^+2^, the mercapto moiety (RS^−^) chelates the zinc ions of the CA active site, leading to its effective inhibition. This study exhibited two of the most potent inhibitors, compounds **26** and **28** (Figure 12), bearing phenylazomethine and a 3-methoxyphenylazomethine functional group with nanomolar IC_50_ values (21.1 and 34.5 nM, respectively). Other molecules from this class, such as **27**, aliphatic derivative **31**, and isatin analogues **32** and **33**, also had a considerable inhibition constant (Figure 12). However, all those antitrypanocidal agents demonstrated poor selectivity indexes with a high possibility of multiple off-target effects. Therefore, all the compounds were proven active ex vivo in *T. cruzi* epimastigotes, and, most importantly, compound **26** was proven active against all *T. cruzi* strains at lower concentrations [115,121,122,123].

A later study by the same group reported the incorporation of halogens and methoxy and phenacetamido groups in heterocyclic sulfonamides. Compounds **34** and **35** stood out with exceptional inhibition constants (0.5–12.5 nM) and demonstrated great selectivity indexes. However, even compounds **34b** and **34f**, that were > 100 fold more selective than their congeners, and they were inactive ex vivo (Figure 12) [118]. This permeability obstacle led the more recent studies towards hydroxamates [115].

**Figure 13:**
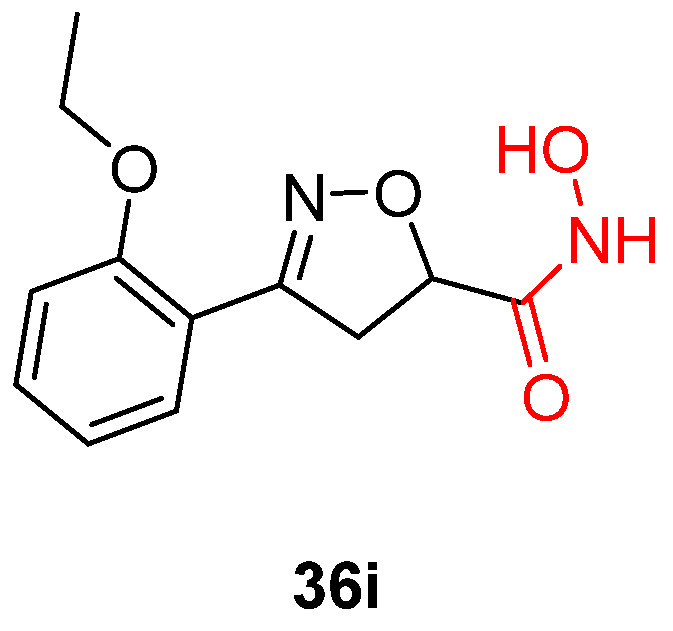
Hydroxamic acid analogue with significant potency and selectivity for *T. cruzi* parasites was found to be more active than clinical drug benzidazole. Metal-chelating atoms/groups are highlighted in red.

A study involving a series of novel 4,5-dihydroisoxazoles introduced hydroxamic acid moieties that could bind with zinc ions. It is already established that hydroxamic acid units are capable of metal coordination in metalloenzymes such as TcCA [124,125]. Among them, compound **36i** (Figure 13) was evaluated in detail and showed promising results both in vitro and ex vivo. Exhibiting a nanomolar IC_50_ value and an S.I value of 6.7, this compound is more potent than benzidazole, the most commonly used drug for the treatment of Chagas disease [115,126]. In order to evaluate its exact mechanism of action, the same authors reported that removing the hydroxamic moiety resulted in the complete loss of antitrypanocidal properties, validating the metal chelation mechanism as a valuable strategy for targeting *T. cruzi* [117,119].

Back in 2011, a novel scaffold was reported that inhibits both the *T. cruzi* and *T. brucei* species [127]. Spiro carbocyclic 2,6-DKP scaffolds attached by the acetohydroxamic unit have already been useful against *T. brucei* parasites.

**Figure 14:**
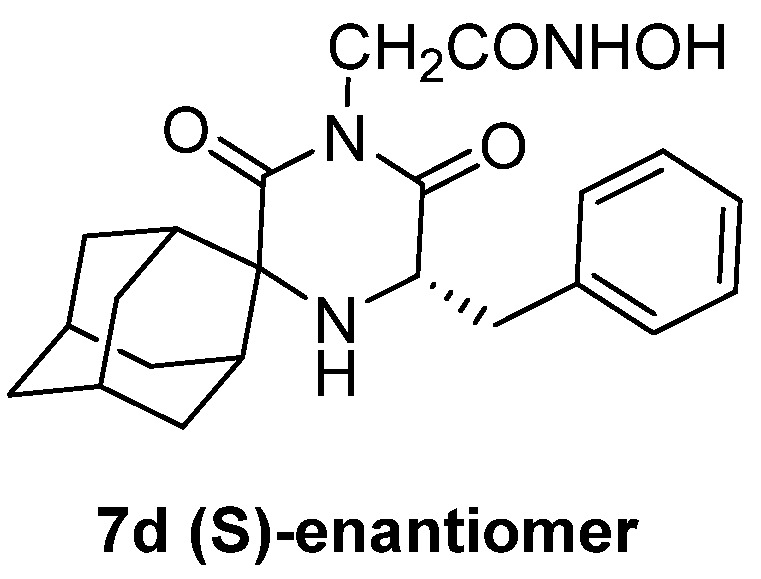
Compound **7d** was proven to be the most active among all of the compounds tested, with an IC_50_ = 0.21 μΜ.

The hydroxamic moiety acts as a metal-chelating functional group and, as a result, it binds to the zinc ion of the catalytic site of the *T. cruzi* metalloenzyme, inhibiting the proliferation of culture cells in vitro [87]. The first active molecule against *T. cruzi* parasites in bloodstream form was compound **7d** (Figure 14), with an IC_50_ value of 0.21 μΜ and a great selectivity index. This compound was also potent against *T. brucei* (IC_50_ = 6.8 nM), demonstrating a possible universal antiviral agent against the two trypanocidal species [127].

**Figure 15:**
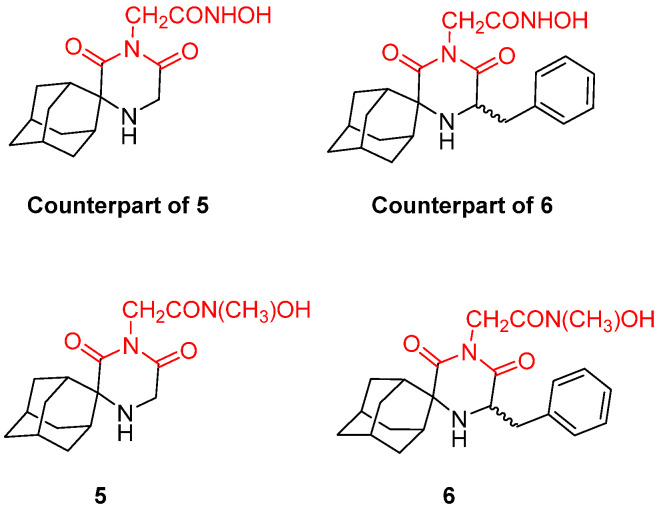
Novel secondary hydroxamic acid derivatives and their potent counterparts. Metal-chelating atoms/groups are highlighted in red.

The above-mentioned compounds constitute primary hydroxamic acid derivatives with proven trypanocidal activity. The following research led to the evaluation of two novel secondary hydroxamic acids **5** and **6** (Figure 15). To evaluate the exact stereoelectronic requirements for the optimization of potency, these two molecules were tested against both *T. cruzi* and *T. brucei*, focusing on their E/Z conformations and their significance for optimal trypanocidal activity. The results were rather disappointing, as these analogues showed decreased potency compared to their unmethylated derivatives. Thus, the presence of two conformers is unfavorable for trypanocidal activity, and future experiments should focus on primary hydroxamic acids [128].

**Figure 16:**
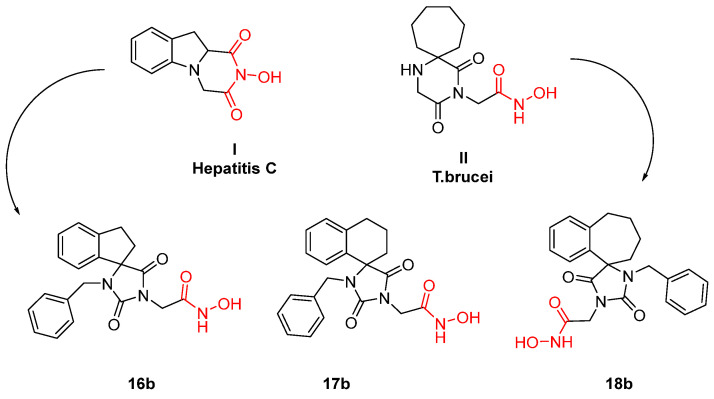
Metal chelators with dual activity, both trypanocidal and antiviral. Metal-chelating atoms/groups are highlighted in red.

In 2019, a chimeric molecule was reported resulting from a combination of an antiviral indole–flutimide analogue and a cycloheptane spiro-substituted 2,6-DKP analogue proven to be potent against trypanocidal species. The idea was to develop a novel antiviral agent capable of inhibiting both HCV and Trypanosome growth. Especially for *T. cruzi* parasites, compounds **16b**, **17b,** and **18b** (Figure 16) were the most promising ones. All three have a benzyl functional group responsible for their potency. In the absence of this moiety, all three molecules are inactive. The calculated EC_50_s ranged from 1.70 μΜ for **16b** to 0.49 μΜ for the bulkier analogue **18b**. These results indicate the same assumption as for the *T. brucei* species: increasing lipophilicity and bulkiness leads to an unexpected increase in trypanocidal potency. This study constitutes the beginning of an unexplored field in which antiviral agents with dual activity are the leads of the unexploited pursuit of a universal antiviral agent that acts on any viral or parasitical metalloenzyme [87].

Apart from hydroxamic acid derivatives, several studies highlight the antitrypanocidal properties of the inorganic anions that combine hydroxamic acid with the sulfonamide scaffold. Alafeefy et al. were the last ones reporting quinazoline sulfonamide analogues with in vitro trypanocidal efficiency that led them to the anion hypothesis [129]. Sulfamic acid, sulfamide, and phenylarsonic and phenylboronic acid are able to inhibit metalloenzymes such as TcCA. Examining other ions, such as iodide, trithiocarbonate, thiocyanate, and hydrogen sulfide, led to the conclusion that this type of anion could become the lead for the design of novel molecules, which could inhibit CA with a different mechanism of action. Diethyldithiocarbamate was found to be the most promising among them [130].

The dithiocarbamate family was first reported by Debus in 1850 [131] for its antiparasitic and antiviral properties. This scaffold is known for its ability to chelate metal ions and consequently inhibit the normal activity of metalloenzymes such as CA. Compared to benzidazole, these compounds showed antiparasitic properties against *T. cruzi* species, which are believed to be due to the formation of metal complexes with the zinc ions located in the CA catalytic site. Through this chelation mechanism, CA loses its functionality, and therefore any repair mechanism of oxidative stress damage is disabled. None of the reported molecules have any further in vitro evaluation so far, opening the way for a new research field on trypanocidal drugs [132].

**Figure 17:**
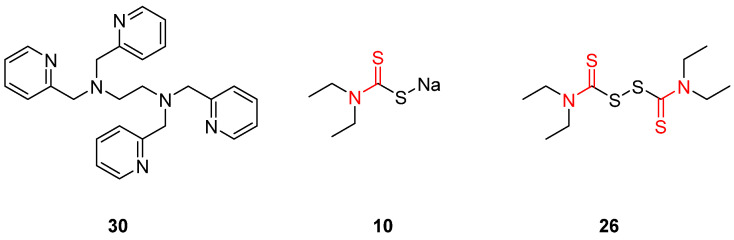
Best dithiocarbamate derivatives acting as metal chelators for zinc-, iron-, and copper-dependent *T. cruzi* enzymes. Metal-chelating atoms are highlighted in red.

Since *T. cruzi* has many more metalloenzymes apart from CA, including copper- and iron-dependent enzymes, a variety of molecules have been examined for their trypanocidal potency. Based on the resulting data, it can be inferred that the compound’s ability to chelate metals, a significant degree of lipophilicity, and specific conformational requirements are critical factors for its potential as a potent trypanotoxic agent. Compound **30** (Figure 17) is one of the most effective metal chelators and was evaluated in a study by Rodriguez et al. with 4-pyridylmethyl moieties even though it does not have any ionically charged groups. The significant antitrypanocidal potency of this molecule is related to its combination of high lipophilicity and its ability to form stable metal complexes providing cell permeability and resilient zinc, copper, or iron bonds. In general, this study proved that any lipophilic substituent incorporated in the nitrogen moiety of dithiocarbamate (NCS_2_Na) combined with a branched side chain leads to effective trypanocidal inhibitors. Moreover, increased hydrophilicity results in the complete loss of activity, illustrating the importance of a certain degree of lipophilicity in the candidate agent. A series of piperidine dithiocarbamates displayed similar inhibition constant values. However, no other molecule demonstrated a pharmacological profile as promising as compound 30, which rapidly penetrates biological membranes and acts only through the metal chelation mechanism. While exhibiting remarkable efficacy against *T. cruzi*, compound **30** is associated with a significant degree of toxicity towards normal cells, thereby possibly limiting its clinical applicability [133].

Another class of metal chelator inhibitors against *T. cruzi* is thiuram disulfides, which are believed to inactivate the superoxide dismutase enzyme by binding to its essential metal ion. Compounds **10** and **26** (tetraethylthiuram disulfide and sodium diethyldithiocarbamate, Figure 17) are the most promising of this class. They have been tested for years against other parasites and diseases, and they are readily available. Focusing on their mode of action and their metal-chelating properties could furnish a basis for the design and optimization of novel antiparasitic drugs targeting *T. cruzi* epimastigotes [133].

## 7. Human Immunodeficiency Virus (HIV) Integrase and Ribonuclease

Human immunodeficiency virus (HIV) is the virus accountable for causing the commonly named acquired immunodeficiency syndrome (AIDS), which has become a major epidemic [134]. It is a severe public health issue, infecting up to 30 million people worldwide [135]. It is widely known that HIV is transmitted mainly through blood and, in most cases, through body fluids during sexual intercourse or that it is transmitted accidentally by blood transfusions or needle sharing. It is still possible for a child to become HIV positive as he can inherit it perinatally from an infected mother [136]. Symptoms vary according to the severity of the HIV disease, and these range from fever, headache, and joint pain to herpes zoster, pneumonia, and cancer, which can lead to death if not treated in time [136]. The current antiviral therapy carries many risks and causes chronic toxicity, therefore the development of new antiviral agents against HIV is important [135]. Thus, synthesizing single molecules targeting two proteins (dual inhibitors) is the main goal in the development of a therapy [135].

The current treatment of HIV infections involves five types of available direct-acting antiviral compounds which are classified according to their mechanism of action. Nucleos(t)ide reverse transcriptase inhibitors (NRTIs) make up the first category of these compounds, represented by zidovudine, the initial licensed anti-HIV drug. Zidovudine is phosphorylated to its active triphosphate form by intracellular kinases, and it competes with deoxythymidine triphosphate (dTTP) and simultaneously blocks the viral DNA’s synthesis by inhibiting the HIV reverse transcriptase [136,137]. Nonnucleoside reverse transcriptase inhibitors (NNRTIs), namely, efavirenz, nevirapine, and etravirine, bind to the HIV reverse transcriptase allosterically and therefore inhibit DNA synthesis [136,138,139]. Protease inhibitors, such as saquinavir, act by obstructing the active site of the HIV protease, thereby hindering the cleavage of the precursor viral proteins [140]. Integrase strand transfer inhibitors (INSTIs), such as raltegravir, inhibit the transfer of the created viral DNA into the host cell genome while blocking the HIV integrase [136]. The most recent class of anti-HIV drugs comprises entry/fusion inhibitors, which are exemplified by enfuvirtide and maraviroc. Enfuvirtide inhibits the fusion of HIV gp41 with the host cell membrane, whereas maraviroc targets the CCR5 membrane receptor that is essential for HIV’s entry into the host cell [136,141,142].

More recent studies have identified that HIV RNaseH shows many similarities with the HBV RNaseH. The former disposes a highly conserved and essential DEDD motif consisting of four carboxylate residues in order to chelate with two divalent metal cations (see Section 3) [57]. Similar to the approved integrase (IN) inhibitors, all the synthesized compounds possess a three-oxygen pharmacophore, which is necessary for metal chelation [57]. These compounds include DKAs, α-HTs, and *N*-hydroxyimide derivatives, which exhibit dual activity, inhibiting both the HIV IN and HIV RNaseH [57].

DKAs were originally detected as Mg^2+^ chelators in the influenza virus endonuclease’s active site. Afterwards, they were identified as metal-chelators in the HIV IN active site and were later tested against HIV RNaseH [134,143]. Since HIV RNaseH and HIV IN display marked similarities, DKAs are often inhibitory compounds against both. Many of them are pyrrolyl DNA derivatives containing a quinolinonyl- or a pyrrolyl-based scaffold reported as dual inhibitors [144,145,146]. Significantly, **RDS1759** (Table 2) was the first selective HIV RNaseH bearing a chlorine atom at position 2 of the benzyl group, and its IC_50_ value is 7.3 μM [57]. Compound **6f** (Table 2) is a derivative of **RDS1759** characterized as a HIV RNaseH and HIV IN dual inhibitor, with an IC_50_ value of 1.8 μM and 1.2 μM, respectively [146]. Quinolinonyl DKA derivatives were further defined as HIV IN inhibitors, and the general characteristics of them are a quinolinone ring disposing a p-fluorobenzyl group as a N substituent; a DKA group (acid or ester) at position 3, with the acid derivatives being more active; and an alterable basic moiety at position 7 [145]. Compound **7b** (Table 2), containing of a pyrrolidine group at position 7, showed an IC_50_ of 5.1 µM when blocking the HIV RNaseH and an IC_50_ of 0.028 µM against HIV IN. Compound **12d** (Table 2), consisting of an *N*-acetyl piperazine instead of the pyrrolidine in the quinolinone ring, demonstrated potent inhibition capacity against both HIV RNaseH (IC_50_ = 3.3 µM) and HIV IN (IC_50_ = 0.08 µM) [57].

Among α-HTs, **β-thujaplicinol** (also discussed in Section 3) has been identified as the most potent HIV inhibitor [147]. In their publication, Chung et al. delineated a series of 14 artificially synthesized derivatives of manicol of which compound **1** (Table 2) manifested the most advantageous characteristics, with an IC_50_ of 0.82 µM against RNaseH [148]. Pyrimidine and hydroxypyridone carboxylic acids contain hydroxypyrimidine or hydroxypyridone rings embedded with DKA moieties as chelating groups [57]. Later, Summa et al. proved that pyrimidinol carboxylic acids are stable substitutes for α,γ-DKAs, and the most potent compound of this series is compound **11** (Table 2), with an IC_50_ = 0.18 µM against HIV RNaseH [149,150]. Compound **10r**, bearing a naphthyl group at N-1, is an effective inhibitor of RNaseH, with an IC_50_ = 2.7 μΜ. Nevertheless, the addition of a 4-sulphonamide diphenyl group at the N-1 position in compound **10y** increased the inhibitory activity significantly, with an IC_50_ value of 0.65 μΜ [57].

*N*-hydroxyimide derivatives are highly potent, and the prototypic *N*-hydroxyisoquinolinedione (HID) (Table 2) especially exerts the low micromolar inhibition of HIV RNaseH activity. The observation that the substitution of the hydroxyl group by either methoxy or amino groups results in the loss of activity underscores the importance of a chelating moiety incorporated into a ring with fixed angles, providing a stable anchor to the active site, and of aromatic substitutions to enhance target affinity. These findings suggest a simple pharmacophoric requirement for HID-based RNaseH inhibitors [57]. Thus, several compounds were outlined as very active RNaseH and IN blockers, such as 2-hydroxy-4-methoxycarbonylisoquinoline-1,3(2H,4H)-dione (Table 2), which displayed an IC_50_ = 0.061 µM against RNaseH and IC_50_ = 4.7 µM against IN [151]. Later on, HID derivative **20i** (Table 2), possessing a biaryl group attached to the chelating core by a single atom linker, was determined to exhibit potency against RNaseH, with an IC_50_ = 0.8 µM [152]. **YLC2-155** (Table 2) is a compound with a similar structure and similar therapeutic efficacy with the only difference being the biaryl moiety’s replacement with a furan structure [153] from which compound **11d** (Table 2) arises as a dual inhibitor of both HIV RNaseH and IN with an IC_50_ = 0.04 μΜ and an IC_50_ = 2.1 μΜ, respectively [57,154].

*N*-hydroxypyrimidinediones (HPDs), particularly compound **45** (Table 2), consisting of a carboxamide and a small alkyl group at positions C-5 and C-6, respectively, demonstrated the highest potency as dual inhibitors of HIV RNase H and IN, exhibiting IC_50_ values of 0.029 μΜ and 0.021 μΜ, sequentially [57]. After several SAR studies, the HPD scaffold was modified with the aim of discovering RNaseH inhibitors with a greater specificity. This involved the elimination of the N-1 and C-3 substituents and the incorporation of a biaryl group at the C-6 position using a flexible linker comprising of a single atom (Table 2) [155,156]. The inhibitory activity in vitro and in cellular assays appeared to be significantly affected by the highly adaptable one-atom linker present at C-6, and the most active compound was **13j** (Table 2), as it inhibited both the RNaseH with an IC_50_ of 0.005 μM and IN with an IC_50_ = 4.0 μM [57]. HNO was identified as the first bicyclic system with inhibitory abilities, with compound **MK-1** (Table 2) being able to block the RNaseH (IC_50_ = 0.11 μΜ) and a 4-p-aminomethyl-biphenyl analog, namely, compound **13** (Table 2), which is selective and very active against both the RNaseH (IC_50_ = 0.045 μM) and IN (IC_50_ = 24 μM). However, the most potent compound representing the HNO scaffold is **XZ460** (Table 2), with a therapeutic index of 26 [57].

*N*-hydroxypyridopyrimidinones and *N*-hydroxypyridopyrazines are two of the most significant categories of HIV inhibitors. More specifically, compound **22** (Table 2), bearing an aminomethyl-diphenyl substituent at position 5, illustrated nanomolar activity against RNaseH (IC_50_ = 10 nM) and micromolar activity against IN (IC_50_ = 0.6 μΜ), and compound **7a** (Table 2) displayed blocking activity against RNaseH with an IC_50_ of 1.77 μM, showing similar potency against IN (IC_50_ = 1.18 μM) [57]. Several *N*’-acylhydrazones are also categorized as dual inhibitors of HIV RNaseH and HIV IN, as they contain functional groups able to chelate the Mg^+2^ cations present in both enzymes’ active sites [135]. Importantly, compound **18** (Table 2) exhibited dual RNaseH and IN blocking activity, with an IC_50_ = 1.7 μΜ and an IC_50_ = 16 μΜ, sequentially [135].

**Figure 18:**
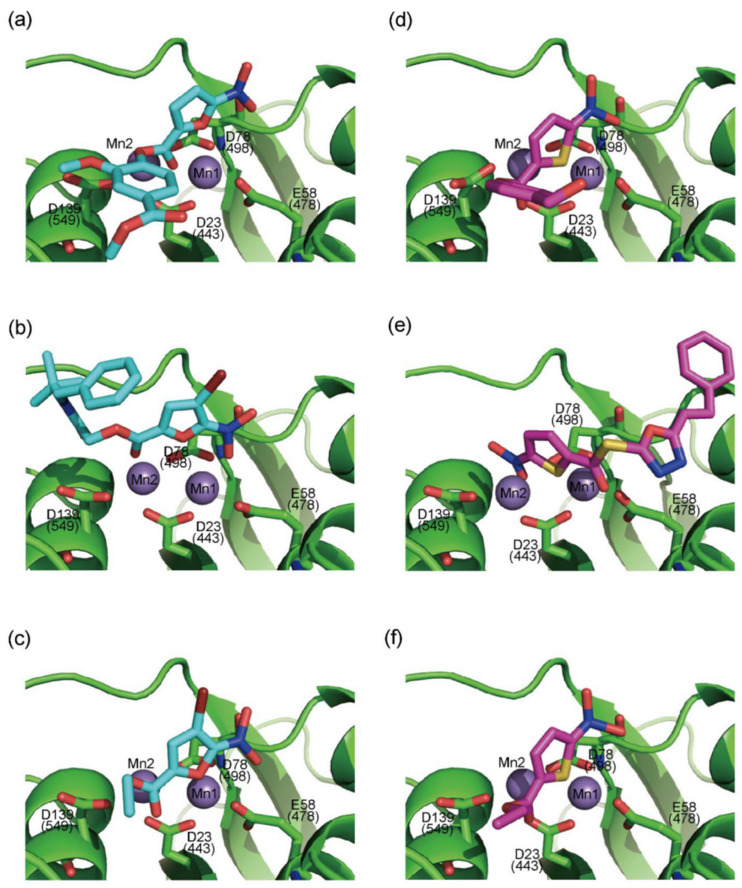
Crystal structures of the complex of RNaseH active site and compounds (**a**–**f**). Mn^2+^ ions are presented as spheres in purple. Reprinted/adapted with permission from Ref. [160]. Copyright © 2023, American Chemical Society.

In 2009, Fuji et al. screened 20,000 compounds and reported that the derivatives of the 5-nitro-furan-2-carboxylic acid carbamoylmethyl ester (NACME) moiety can inhibit HIV-1 RNaseH activity. Two of these derivatives, namely, compounds **1** and **2** (Table 2), specifically inhibited the HIV-1 RNaseH with IC_50_ values in the low μM range without affecting the RT polymerase or IN activity. Although no cytotoxicity was observed for compound **1**, compound **2** exhibited cytotoxicity at 30 μΜ or more. The main limitation of this class of inhibitors is their limited efficacy in cell assays, probably due to low membrane permeability (many polar groups are present) or decreased stability [157]. The 5-intro-furan-2-carboxylic acid scaffold was further explored through various modulations of parent compounds **1** and **2** in order to establish a more precise SAR model. This campaign proved that the nitro-furan group is indispensable due to its antiviral activity and can only be changed with thiophene, as the oxygen (and/or sulfur) atoms present in the core ring and the ester linkage chelate the divalent cations of the active site as proven by enzymatic experiments and computational theoretical calculations. Various substitutions are tolerated in the side chain without the loss of the inhibitory activity, with the most potent compounds bearing a phenyl-ester group connected to the nitro-furan ring and also methoxy-carbonyl and methoxy groups on the phenyl ring (compounds **a**–**f**, Table 2). Importantly, all the synthesized compounds showed no cytotoxicity [158,159]. Six of these newly synthesized compounds were cocrystallized with a recombinant partial protein comprising of the HIV-1 RNaseH active site. The results indicated very different binding structures between the analogs bearing the furan and thiophene ring. Even though metal chelation is strong in both compound types, the metal ions are located in the same plane, with the O containing the furan ring (Figure 18a–c), whereas they move out of the core ring plane when S is present instead of O (Figure 18d–f) [160].

## 8. Conclusions

In summary, metalloenzymes play a vital role in the replication cycle of both viruses and parasites. However, their inhibition by small molecules that block their activity though chelation with metal atoms, which are necessary for their catalytic activity, still remains a rather unexplored space. Recent efforts have managed to shed more light in the field of metalloenzyme inhibition, although only a small number of molecules has managed to enter clinical trials let alone achieve market approval. The biggest limitation of the novel metalloenzyme inhibitors is their very limited cell permeability and high hydrophilicity due to the existence of the metal-chelating groups, which all contain oxygen or nitrogen atoms. It is therefore evident that research efforts should focus on addressing the hydrophilicity issue and, at the same time, the improvement of the key interactions within the binding site with the aim of achieving even greater potency and selectivity.

Besides their limitations, the metal-chelating inhibitors of viral and parasitic metalloproteins constitute very attractive, promising, and safe therapeutic agents for the treatment of serious viral and parasitic diseases. Additionally, it is noteworthy that, with fine structural tuning, these agents could serve as dual target inhibitors, thus remaining one of the hottest topics in drug discovery. This very intriguing approach, followed by our lab over the last 10 years, attracts the pharmaceutical industry’s interest, as it can potentially limit the number of administered drugs, their chronic toxicity, and the possibility of developing drug-resistant viruses and parasites.

## Figures and Tables

**Table 1:** HBV ribonuclease H inhibitors. The highlighted red atoms are the atoms responsible for cheating the two Mg^2+^ ions in the enzymes’ catalytic sites. HID represents *N*-hydroxyisoquinolinedione, HNO represents *N*-hydroxynaphthyridine, HPD represents *N*-hydroxypyridinedione, EC_50_ represents half-maximal effective concentration, CC_50_ represents 50% cytotoxic concentration, and TI represents therapeutic index (TI = CC_50_/EC_50_).

α-Hydroxytropolones					
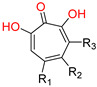 **α-hydroxytropolones**	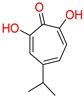 **β-thujaplicinol** [60] **EC_50_ = 1.0 μΜ** **CC_50_ = 25 μΜ** **TI = 25**		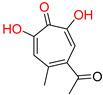 [60] **EC_50_ = 0.34 μΜ** **CC_50_ = 32 μΜ** **TI = 94**		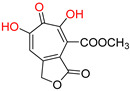 [59] EC_50_ = 0.5 μΜ CC_50_ = >77 μΜ TI = >154
***N*-hydroxyimides**					
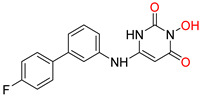 [48] HID EC_50_ = 1.4 μΜ CC_50_ = 99 μΜ TI = 71		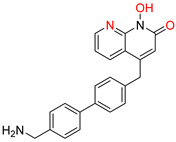 [55] HNO EC_50_ = 3.4 μΜ CC_50_ = 7.1 μΜ TI = 2		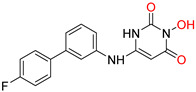 [55] N-hydroxypyrimidinedione EC_50_ = 5.5 μΜ CC_50_ = >100 μΜ TI = >18	
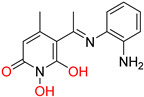 **208** [48] HPD EC_50_ = 0.69 μΜ CC_50_ = 15 μΜ TI = 22		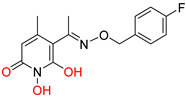 **A23** [56] HPD EC_50_ = 0.11 μΜ CC_50_ = 33 μΜ TI = 300		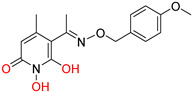 **A24** [56] HPD EC_50_ = 0.29 μΜ CC_50_ = 102 μΜ TI = 352	

**Table 2:** Structures of reported HIV integrase and ribonuclease H inhibitors. Metal-chelating atoms/groups are highlighted in red.

Diketo Acids (DKAs)			
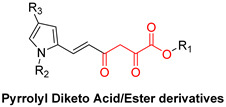	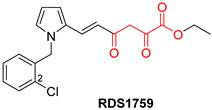		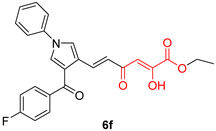
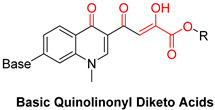	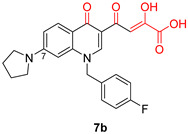		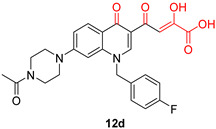
**α-hydroxytropolones**			
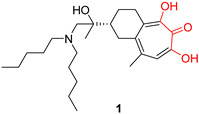			
**Hydroxypyrimidine and hydroxypyridone carboxylic acids**			
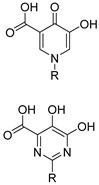	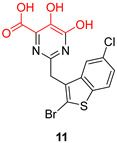		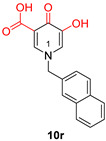
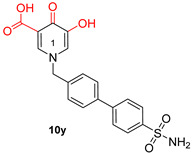			
***N*-hydroxyimides**			
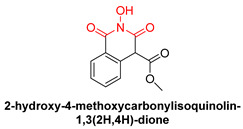	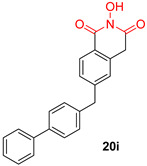		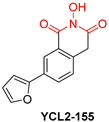
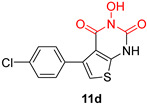			
***N*-hydroxypyrimidinediones (HPDs)**			
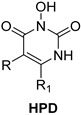	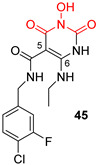		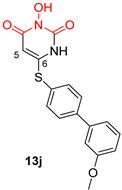
***N*-hydroxynaphthyridine (HNOs)**			
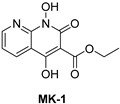	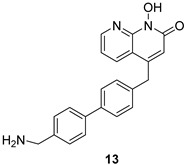		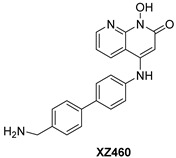
***N*-hydroxypyridopyrimidinones**		***N*-hydroxypyridopyrazines**	
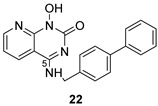		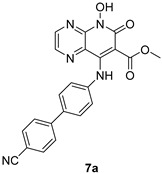	
***N*′-acylhydrazones**			
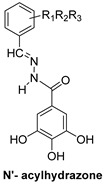		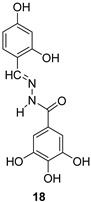	
**5-nitro-furan-2-carboxylic acid derivatives**			
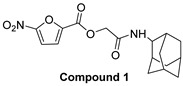		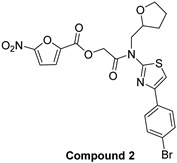	
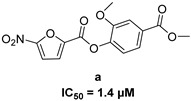	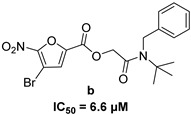		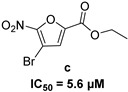
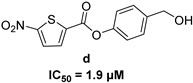	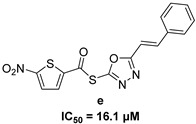		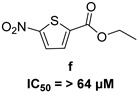

## Data Availability

Not applicable.
